# The impact of high-intensity interval training on cerebrovascular function in the APP/PS1 mice

**DOI:** 10.3389/fragi.2025.1647628

**Published:** 2025-10-14

**Authors:** Lei Zhu, Ming Cai, Zhe Lu, Qi Wang, Teng Zhai, Jingyun Hu

**Affiliations:** ^1^ College of Sport Sciences, Qufu Normal University, Qufu, Shandong, China; ^2^ College of Rehabilitation Sciences, Shanghai University of Medicine and Health Sciences, Shanghai, China; ^3^ Central Lab, Shanghai Key Laboratory of Pathogenic Fungi Medical Testing, Shanghai Pudong New Area People’s Hospital, Shanghai, China

**Keywords:** high-intensity interval training, Alzheimer’s disease, pathological biomarkers, neuroplasticity, cerebrovascular function

## Abstract

**Abstract:**

Alzheimer’s disease (AD), the most commonly diagnosed form of senile dementia worldwide, is closely associated with aging and distinct neuropathological features. Recent studies highlight that up to 90% of individuals, either preclinical or clinical, diagnosed with vascular pathology in the context of AD exhibit thickening and hyalinization of the media in small and medium-sized cerebral vessels. Exercise has emerged as a potential, non-pharmaceutical, and cost-effective intervention for the prevention and treatment of AD. However, there is limited research exploring the effects of high-intensity interval training (HIIT) on cerebrovascular function in AD.

**Methods:**

Four-month-old female C57BL/6 J mice and APP/PS1 transgenic mice were initially acclimated to a standard diet for 1 week. The two groups were then divided into sedentary and exercise cohorts, with the exercise group engaging in a 6-week HIIT regimen. Post-intervention, hippocampal specimens were collected for analysis. Aβ and Tau protein levels were measured to assess AD pathology, while cognitive function was evaluated using the eight-arm radial maze and BDNF mRNA expression. Additionally, markers of cerebrovascular function-including VEGF, EPO, eNOS, GPR68, and ET-1-were examined, and HIF-1α was utilized to assess the hippocampal response to AD pathology.

**Results:**

HIIT significantly reduced reference memory errors (*p =* 0.025) and markedly upregulated *Bdnf* mRNA expression *(p <* 0.001) specifically in APP/PS1 mice. Furthermore, HIIT significantly decreased protein levels of AD pathological markers p-TAU (*p =* 0.001) and APP (*p =* 0.002) in APP/PS1 mice. HIIT significantly increased the mRNA (*p <* 0.001) and protein (*p =* 0.003) levels of EPO and *Vegfa* mRNA (*p <* 0.001) levels to stimulate pro-angiogenic signal in APP/PS1 mice. HIIT also significantly increased both the mRNA and proteins levels of eNOS expression (*p <* 0.001) while decreasing the mRNA and proteins levels of ET-1 (*p* < 0.001) and GPR68 (*p <* 0.001) to enhance vasodilation in APP/PS1 mice. Finally, HIIT significantly increased HIF-1α expression at both protein and mRNA levels (*p <* 0.001), independent of genotype.

**Conclusion:**

HIIT ameliorates cognitive function and reduces hallmark AD pathology. This positive effect is potentially mediated through cerebral microangiogenesis, cerebrovascular function regulation, and hypoxic metabolism. HIIT represents a promising non-pharmacological strategy for targeting multiple aspects of AD pathophysiology.

## 1 Introduction

Alzheimer’s disease (AD) is the most common cause of dementia globally, with its prevalence increasing alongside global aging (Weller and Budson, 2018). This progressive neurodegenerative disease is characterized by memory loss and multiple cognitive impairments. In the earliest phases of AD, accumulated amyloid β (Aβ) deposits on the walls of cerebral vasculature, coupled with the spread of tau pathology, are observed (Scheltens et al., 2021; Wójtowicz et al., 2020). A promising treatment strategy in clinical trials is poised to develop the anti-amyloid β, anti-tau, and anti-inflammatory targeted drugs (Scheltens et al., 2021). As individuals age, central arterial stiffness and increased arterial pulsation are associated with higher cerebrovascular resistance, reduced cerebral blood flow, and cerebrovascular dysfunction, which are major risk factors for dementia (Tomoto and Zhang, 2024). Notably, 80%–90% of preclinical or clinical people have been diagnosed cerebral vascular amyloidosis (CAA) via neuroimaging technique (Dorner et al., 2024; Charidimou et al., 2022), characterized as one of the biomarkers of AD, before the development of classical significant cognitive decline (Graff-Radford et al., 2021). In the rodent AD model, previous research has revealed that early morphological abnormalities (Zhang et al., 2018; Lifshitz et al., 2012) and microvascular degeneration spread in the subregions of the hippocampus and cortex via the whole-brain analysis of the angioarchitecture (Quintana et al., 2021). Furthermore, declines in regional cerebral blood flow (CBF) and diminished functional and structural connectivity have been observed, both linked to cerebrovascular function (Wiesmann et al., 2017). Evidence suggests that vascular and endothelial dysfunctions play critical roles in the progression of AD, significantly exacerbating underlying neurodegenerative processes (Palmer et al., 2025; Stelzer and Lima-Filho, 2024).

Erythropoietin (EPO) and vascular endothelial growth factor (VEGF) are two vasoactive molecules crucial for brain development due to their trophic effects. These molecules respond to neuronal damage and confer neuroprotective and neurorestorative benefits to the central nervous system, and mitigate brain injury (Urena-Guerrero et al., 2020; Shim and Madsen, 2018). In the context of the brain, these molecules primarily contribute to cell survival, neural regeneration, neurogenesis, and vascular remodeling (Shibuya, 2014; Kaur et al., 2022), suggesting potential novel therapeutic targeted strategies for AD treatment. Endothelial nitric oxide synthase (eNOS) is a calmodulin-dependent enzyme that produces nitric oxide (NO). This signaling molecule plays a crucial role in neuronal survival, synaptic plasticity, vascular smooth muscle relaxation, and endothelial cell permeability in the brain (de la Monte et al., 2000). A deficiency of eNOS can damage the blood-brain barrier (BBB) and cause insufficient mild chronic perfusion, which resulted in cerebrovascular injury in middle-aged mice (Chen X. et al., 2022). G-protein coupled receptor 68 (GPR68), also known as OGR1, functions as a cellular sensor of acidification (Ludwig et al., 2003). It has been reported to be expressed in endothelial cells of small-diameter arteries and serves as the flow sensor (Xu et al., 2018). It is also expressed in the human aortic smooth muscle cells to regulate the signaling of cAMP-cyclooxygenase-2 (COX2)-prostaglandin I2 (PGI2) for vasodilatation (Sisignano et al., 2021). Endothelin-1 (ET-1) is widely distributed in microvascular endothelial cells, neurons, and glial cells in the central nervous system (CNS) (Li et al., 2018). It was identified as the most potent vasoconstrictor peptide with proliferative, prooxidative, and proinflammatory properties (Kuwaki et al., 1997), which reduces CBF (Chen et al., 2013).

Hypoxia-inducible factor-1α (HIF-1α) is a nuclear transcription factor that responds to oxygen availability by binding to hypoxia response elements (HREs) and activating downstream transcription factors (Yang et al., 2022), such as EPO, VEGF, eNOS, GPR68, and ET-1. These factors promote erythropoiesis, angiogenesis, and vasodilation to overcome hypoxia (McLaren et al., 2007). ​​Regarding the regulation of vascular factors, HIF bounds to EPO 5’ hypoxic response element, and EPO gene transcription increases under the condition of anemia or hypoxia (Shih et al., 2018). Inhibiting or interfering with the activation of HIF-1α reduces the expression of VEGF at both the protein and mRNA levels from 6 h onwards (Wang et al., 2019). Transient transfection of HIF-1α in the endothelial cells was reported to result in a 2.7-fold increase in NOS promoter activity (Palmer et al., 1998). ET-1 ​​is​​ a major contributor to vascular inflammatory ​​remodeling​​, likely through HIF-1-dependent activation of NF-κB (Singh and Prakash, 2017). Under hypoxia, HIF-1α can bind to the promoter of GPR81 to increase its activity (de Valliere et al., 2016). Therefore, HIF-1α may be a critical molecule for regulating these factors and cerebral vascular function in AD.

Exercise has been shown to induce beneficial changes in cerebrovascular health (Nishijima et al., 2016; Whitaker et al., 2020). It enhances cerebral blood oxygen supply, improves antioxidant signaling pathways, and increases cerebral blood flow (CBF), thereby potentially enhancing synaptic plasticity and cognition for the treatment and prevention of AD (Bliss et al., 2021; De la Rosa et al., 2020). High-intensity interval training (HIIT) is an exercise pattern of alternating periods of high-intensity aerobic exercise with light recovery exercise or no exercise (Taylor et al., 2019), which is superior to moderate-intensity continuous training (MICT) in cardiometabolic processes and physical performance (Gripp et al., 2021; Miguet et al., 2020). Recent evidences have shown the beneficial effect HIIT on the AD -like pathology. For instance, it promoted Aβ and p-Tau clearance by regulating astrocytic metabolism (Feng et al., 2023) and mitochondrial energy metabolism (Liu et al., 2022). In the aspect of cerebrovascular function regulation, acute HIIT was reported to increase the de/oxygenated hemoglobin and cerebrovascular reactivity and decrease the middle cerebral artery blood and velocity, thereby improving dynamic cerebral autoregulation (Whitaker et al., 2020; Komiyama et al., 2020). Several research also indicate that HIIT ​​better maintains​​ post-exercise CBF and velocity compared to MICT, supporting sustained brain function (Coates et al., 2023; Whitaker et al., 1985). Furthermore, HIIT stimulates cerebral microangiogenesis in wild-type mice​​ (Morland et al., 2017), enhancing cerebrovascular plasticity. Collectively, these findings suggest HIIT may reduce cerebrovascular risk, although little research has reported the role of HIIT in CAA no matter to say the AD-related cerebral pathology. However, the mechanism links between HIIT and cerebrovascular protection remains poorly understood.

In this study, vascular-related factors were explored in the hippocampus to test the hypothesis that HIIT mitigates AD-related pathology by enhancing cerebral microvascular function, with a focus on molecules of angiogenesis (EPO and VEGF), cerebrovascular regulation (eNOS, ET-1, and GPR68), and hypoxic adaptation (HIF-1α).

## 2 Materials and methods

### 2.1 Animals

28 female 4-month-old C57BL/6J and APP/PS1 mice, weighing 20–23 g, were procured from the Shanghai south model organism (China). After 1 week of acclimation the mice were randomly divided into a control group (n = 7), APP/PS1 group (n = 7), control + HIIT group (n = 7), and APP/PS1 + HIIT group (n = 7). Random numbers were generated using the standard = RAND () function in excel. three to four mice per group were housed in IVC cages under controlled conditions (22 C–24 C, 12:12 light-dark cycle) with *ad libitum* access to food and water, supplemented with environmental enrichment. Daily welfare monitoring included assessments of body weight, fur condition, behavior, and social interactions, with predefined humane endpoints (>20% weight loss, severe lethargy, or inability to eat/drink). Following the last behavioral testing, mice were humanely euthanized via sodium pentobarbital (150 mg/kg i.p., in 0.9% saline). The hippocampus was rapidly dissected on a pre-cooled crushed ice box, following standard procedures, and then stored at −80 C. The study complied with ARRIVE Guidelines 2.0, the Institutional Animal Ethics Committee (IAEC), and the Ethical Committee for Science Research at Qufu Normal University (Approval No. 2020-0001). The suffering and number of animals used was minimized without compromising the power of the experimental design.

### 2.2 HIIT paradigm

**FIGURE 1:**
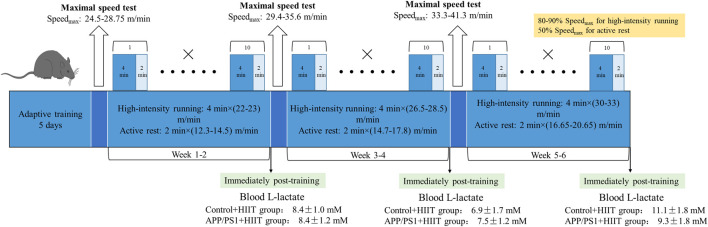
The timeline of HIIT protocol.

Mice were familiarized with five bouts of treadmill running (4 min × (20-22) m/min) and active rest (2 min × 10 m/min) for 5 days and accepted HIIT training. After familiarization, the mice completed a 6-week HIIT. They were exposed to the HIIT exercise protocol for five consecutive days each week. This exercise regime has been previously described (Hu et al., 2021). Each session consisted of warm-up (10 min × 10 m/min), followed by 10 bouts of 4 min high-intensity running (4 min × (80-90)% Speed_max)_, and separated by active rest (2 min × 50 % Speed_max)_. Running took place on a treadmill at a 0°. The maximal speed test for mice was respectively performed at the end of adaptive training, the second and the fourth week of training, and the speed of interval training. The beginning speed of mice was 10 m/min for lasting 10 min to warm up. Then the speed was increased by 2 m/min every 2 min until exhaustion. In the first and second weeks, the HIIT group completed warm-up (10 min × 10 m/min) and then 10 bouts of high-intensity running (4 min × (22-23) m/min), and separated by active rest (2 min × (12.3-14.5) m/min) (the blood L-lactate was 8.4 ± 1.0 mM in control + HIIT group and 8.4 ± 1.2 mM in APP/PS1 + HIIT group immediately post-training). In the third and fourth weeks, the HIIT group completed warm-up and then 10 bouts of high-intensity running (4 min × (26.5-28.5) m/min), and separated by active rest (2 min × (14.7-17.8) m/min) (the blood L-lactate was 6.9 ± 1.7 mM in control + HIIT group and 7.5 ± 1.2 mM in APP/PS1+HIIT group immediately post-training). In the fifth and sixth weeks, the HIIT group completed warm-up and then 10 bouts of high-intensity running (4 min × (30-33) m/min), separated by active rest (2 min × (16.65-20.65) m/min) (the blood L-lactate was 11.1 ± 1.8 mM in control + HIIT group and 9.3 ± 1.8 mM in APP/PS1 + HIIT group immediately post-training) (Figure 1).

### 2.3 Eight-arm radial maze test

**FIGURE 2:**
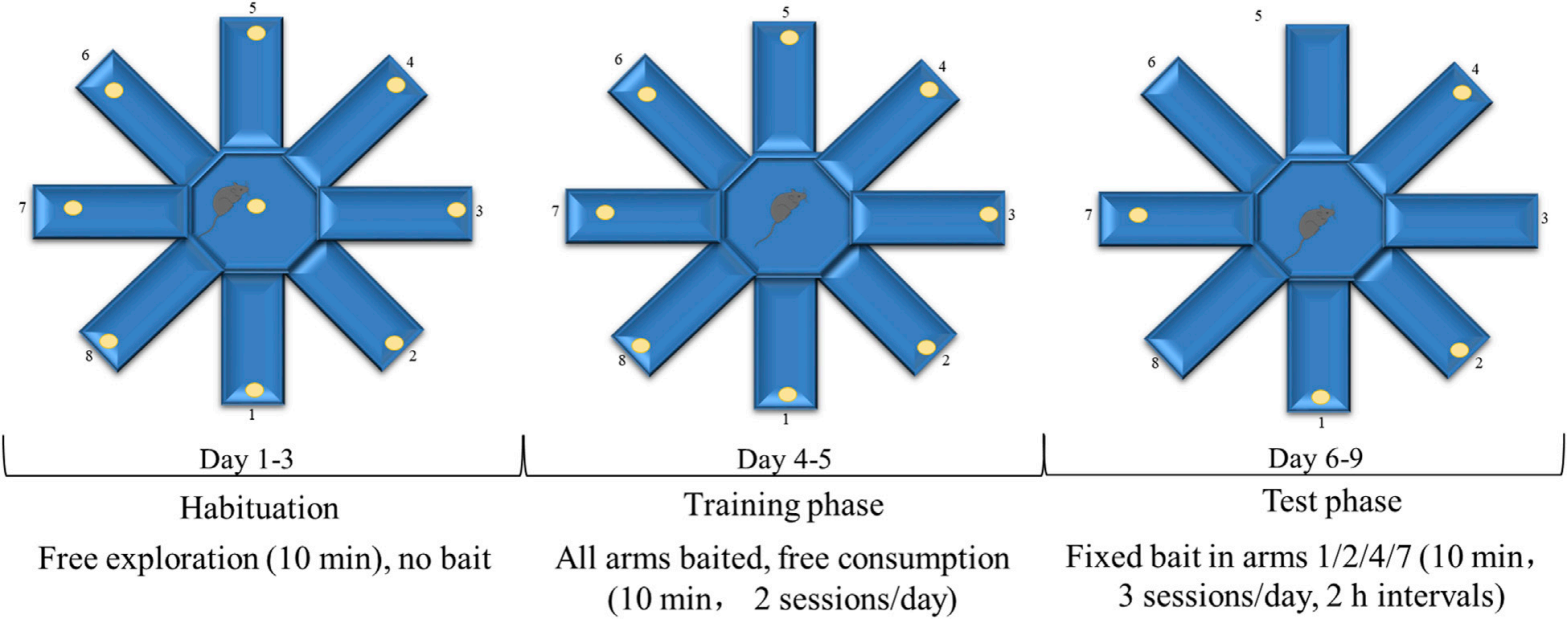
The timeline of eight-arm radial maze test.

At the end of the 6-week training, all mice performed the eight-arm radial maze test to evaluate cognitive function under strictly blinded conditions, with all experimenters unaware of genotype and treatment assignments. Animals were randomized by an independent researcher to maintain blinding integrity throughout data collection and analysis. The radial eight-arm maze ​​apparatus comprised​​ eight ​​sidearms​ (50 cm long × 10 cm wide × 30 cm high) extending from an octagonal central area. 2 days before the test, the mice were weighed and placed on a restricted diet​​ (2–3 g, adjusted based on body weight) daily to maintain their body weights at 85%. The maze consisted of eight arms, and four to five food baits per arm were distributed throughout the arms and central region of the maze. During the initial training period from the first to the third day, the seven animals in each group were allowed unrestricted feeding and free exploration for 10 min in the maze to get familiar with the environment. From the fourth and fifth day, each animal was trained individually. One food bait was placed in each arm and the animal was free to consume as desired for 10 min. From the sixth to the ninth day, one bait was placed in arms 1, 2, 4, and 7. Each mouse was allowed to freely move around the maze and consume the pellets in each arm for 10 min daily. The number of food arms first were entered by the mice without record until re-entering the arm where the bait was previously placed (the total number of entries into baited arms during a single trial - 1), recording as a working memory error. The number of entering the arm where food has not been placed (the total number of entries into arms without food pellets throughout the testing phase) was recorded as a reference memory error. After each test, wipe the inner wall of the maze with 75% ethanol to eliminate residual odor interference. The record was verified by two blinded observers (inter-rater reliability >90%), with maze surfaces sanitized between trials and unmotivated trials excluded per predefined criteria. Three training sessions were conducted each day, with breaks of more than 2 h between sessions (Figure 2). This protocol has been previously described (Braida et al., 2002).

### 2.4 L-lactate measurements

Blood lactate was detected by Lactate-Scout (EKF Co., Germany) immediately at the end of second, fourth and sixth week of training. Seven mice in each group were collected tail tip blood immediately at the end of training.

### 2.5 Western blotting

The hippocampus of each mouse was washed with pre-cooling PBS. The sample was homogenated at 14,000 × g for 5 min in RIPA Lysis Buffer (No. P0013B, Beyotime, Shanghai, China), which contained phosphatase and protease inhibitors, with 1 mM PMSF (No. ST506, Beyotime, Shanghai, China), and the supernatant was collected. Material from vitro experiments was processed similarly. Proteins (30 μg) in SDS were loaded on a 10%–12.5% polyacrylamide gradient gel. The gel was then transferred to a 0.22 μm PVDF membrane (Epizyme Biotech, Shanghai, China). The membranes were blocked with Protein Free Rapid Blocking Buffer (No. PS108P Epizyme Biotech, Shanghai, China) for 10 min, followed by an overnight incubation at 4 °C with gentle shaking using primary antibodies. After washing, the membranes were incubated with secondary antibodies for 1 h at room temperature. The primary antibodies used include HIF-1α (1:1000, No. AF1009, Affinity, USA), EPO (1:1000, No. AF5190, Affinity, USA), GPR68 (1:1000, No. AF0723, Affinity, USA), VEGFA (1:5000, No. 26157-1-AP, Proteintech, USA), ET-1 (1:1000, No. 12191-1-AP, Proteintech, USA), eNOS (1:5000, No. 27120-1-AP, Proteintech, USA), p-TAU (1:1000, No. 28866-1-AP, Proteintech, USA), APP (1:1000, No. 25524-1-AP, Proteintech, USA). Quantification of the band density was performed using ImageJ. Protein levels were normalized to the band intensity of β-ACTIN (1:1000, No. 4970S, Cell Signaling Technology, USA).

### 2.6 RNA extraction and Real-Time PCR (RT-PCR)

Total RNA was prepared from the rested hippocampal tissues using TRIZOL (Ambion, USA). The RNA quality was assessed on a NanoDrop 2000 (Thermo, USA), where the 260/280 ratio was obtained. Samples with a ratio of 1.8–2.0 were processed for gene analysis. Reverse transcription was performed using PrimeScript™RT Master Mix (No. RR036A, Takara) according to the manufacturer’s protocol. Real-time PCR was performed using SYBR^®^ Premix Ex Taq II (No. RR820A, Takara) and StepOnePlus Real-Time PCR System (Applied Biosystems, CA, USA). The mRNA levels of *Bdnf* (Forward TCA​TAC​TTC​GGT​TGC​ATG​AAG​G, Reverse ACA​CCT​GGG​TAG​GCC​AAG​TT), *Epo* (Forward ACT​CTC​CTT​GCT​ACT​GAT​TCC​T, Reverse ATC​GTG​ACA​TTT​TCT​GCC​TCC), *Vegfa* (Forward CTG​CCG​TCC​GAT​TGA​GAC​C, Reverse CCC​CTC​CTT​GTA​CCA​CTG​TC), Nos1 (Forward CTG​GTG​AAG​GAA​CGG​GTC​AG, Reverse CCG​ATC​ATT​GAC​GGC​GAG​AAT), *Gpr68* (Forward TAT​CTT​GCC​CCA​TCG​ACC​ACA, Reverse AGT​ACC​CGA​AGT​AGA​GGG​ACA), *Edn1* (Forward GCA​CCG​GAG​CTG​AGA​ATG​G, Reverse GTG​GCA​GAA​GTA​GAC​ACA​CTC), *Hif1α* (Forward ACC​TTC​ATC​GGA​AAC​TCC​AAA​G, Reverse CTG​TTA​GGC​TGG​GAA​AAG​TTA​GG) were normalized to the internal loading control of *Gapdh* (Forward AGG​TCG​GTG​TGA​ACG​GAT​TTG, Reverse TGT​AGA​CCA​TGT​AGT​TGA​GGT​CA) and determined with the comparative ΔΔCT method.

### 2.7 Statistics

All data are presented as means ± SE. Comparisons of two groups were performed using an unpaired Student’s t-test. A *p* value of 0.05 or less was considered statistically significant. Comparisons of multiple groups were performed using the two-way analysis of variance (two-way ANOVA) with ​​the following main effect: genotype (Control group or APP/PPS1 group) or exercise (APP/PPS1 group or APP/PPS1 + HIIT group), ​​as well as​​ genotype × exercise interaction. If significant interactions were observed, ​​*post hoc*​​ simple effect tests were performed. All calculations and the graph construction were performed using SPSS 20.0 and GraphPad software (La Jolla, CA, USA).

## 3 Results

### 3.1 The effect of HIIT on the cognitive function of APP/PS1 mice

**FIGURE 3:**
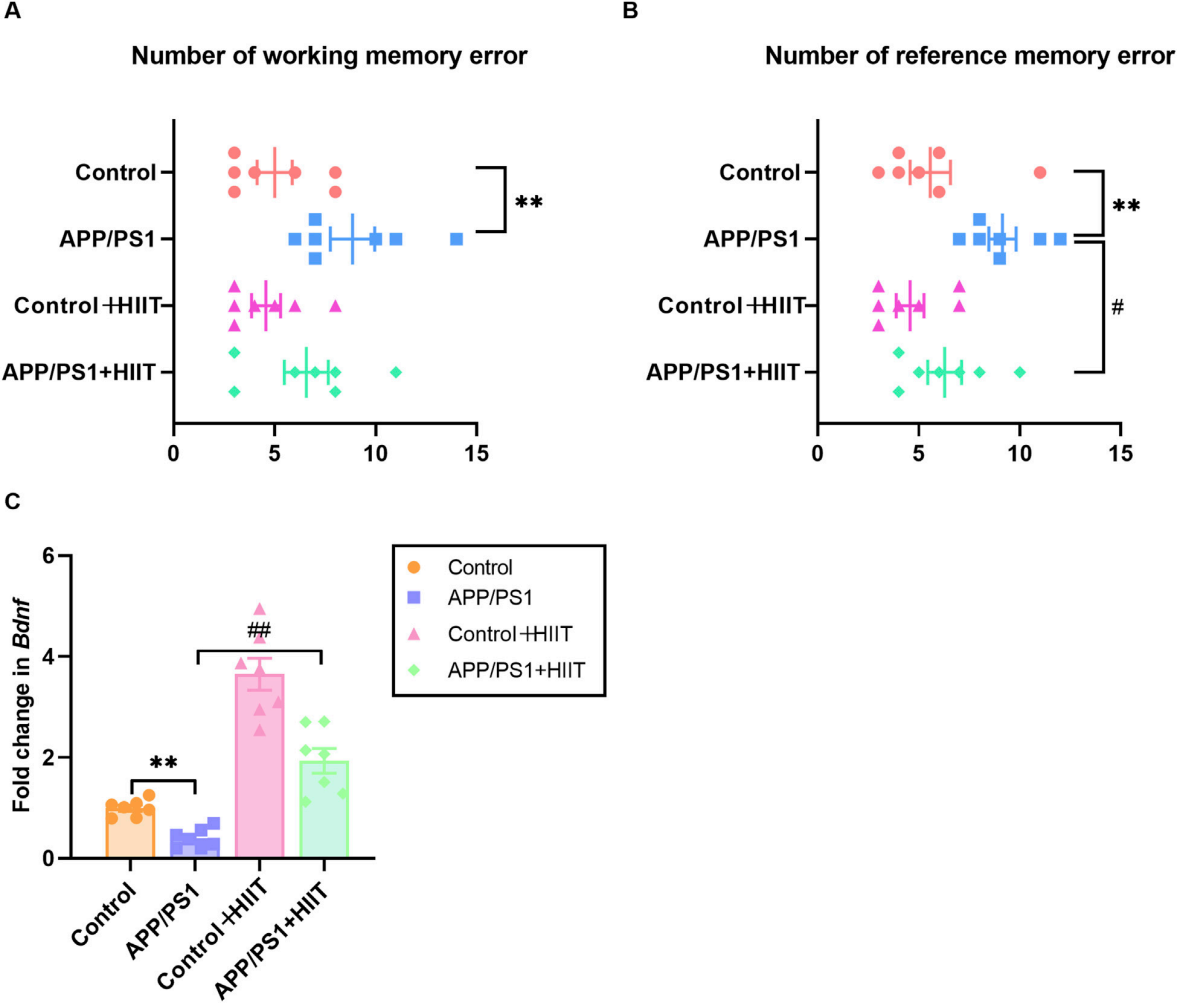
The effect of HIIT on the memory and Bdnf expression in APP/PS1 mice. **(A)** the number of working memory errors in an eight-arm radial maze. **(B)** the number of reference memory errors in an eight-arm radial maze. **(C)** the mRNA expression of *Bndf*. *Gapdh* is an internal control for quantification. Data are displayed as mean ± SE (n = 7). ^*^: *p* < 0.05, *p* < 0.01 APP/PS1 group versus Control group; ^#^: *p* < 0.05, *p* < 0.01 APP/PS1 + HIIT group versus APP/PS1 group.

To evaluate whether HIIT could ameliorate cognitive deficits in APP/PS1 mice, we first assessed spatial memory by using the eight-arm radial maze and synaptic plasticity by measuring the *Bdnf* mRNA levels. The working memory errors in control group, APP/PS1 group, control + HIIT group, and APP/PS1 + HIIT group was 5.00 ± 0.87, 8.86 ± 1.10, 4.57 ± 0.72, and 6.57 ± 1.09, respectively. The reference memory errors in control group, APP/PS1 group, control + HIIT group, and APP/PS1 + HIIT group was 5.57 ± 1.00, 9.14 ± 0.67, 4.57 ± 0.69 and 9.14 ± 0.67, respectively. As shown in Figures 3A,B, the genotype × exercise interaction was not significant in both working memory errors and reference memory errors. The main effect of genotype was significant in working memory errors (F (1,24) = 9.339, *p* = 0.005, η^2^ = 0.280) and reference memory errors (​​F (1,24) = 10.695, *p* = 0.003, η^2^ = 0.308​​). The main effect of exercise was significant in reference memory errors (​​F (1,24) = 5.695, *p* = 0.025, η^2^ = 0.192) but not significant in working memory errors (F (1,24) = 2.006, *p* = 0.170, η^2^ = 0.077).

In terms of *Bdnf* expression, a significant genotype × exercise interaction was observed (F (1,24) = 7.328, *p* = 0.012, η^2^ = 0.234). The main effect of genotype (F (1,24) = 31.406, *p* < 0.001, η^2^ = 0.567) and exercise (F (1,24) = 102.958, *p* < 0.001, η^2^ = 0.811) were significant. Then the simple effect test was conducted. Compared with the APP/PS1 group, HIIT significantly increased the mRNA levels of *Bdnf* (F (1,24) = 31.406, *p* < 0.001, η^2^ = 0.567) (Figure 3C). These results indicate that while HIIT may have limited effects on working memory, it can significantly improve reference memory and upregulate BDNF expression in AD mice, potentially supporting synaptic maintenance.

### 3.2 The effect of HIIT on the pathological markers in APP/PS1 mice

**FIGURE 4:**
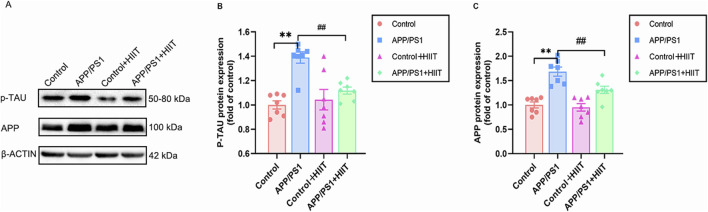
The effect of HIIT on the expression of p-TAU and APP in AD. **(A)** the Western blot image of p-TAU and APP. **(B)** quantification of ​​p-TAU​​ protein expression in each group. **(C)** quantification of ​​APP​​ protein expression in each group. β-ACTIN is a loading control. Data are expressed as mean ± SE (n = 7). ^*^: *p* < 0.01 APP/PS1 group versus Control group; ^#^: *p* < 0.01 APP/PS1+ HIIT group versus APP/PS1 group.

P-TAU and APP have demonstrated specificity to AD versus non-AD neurodegenerative diseases, which were critical for clinical diagnosis and eligibility for therapies. To determine whether cognitive improvements were associated with reduced AD pathology, we examined these two proteins. A significant genotype × exercise interaction was observed in p-TAU (F (1,24) = 8.868, *p* = 0.007, η^2^ = 0.270) and APP (F (1,24) = 4.410, *p* = 0.046, η^2^ = 0.155). The main effect of genotype was significant in p-TAU (F (1,24) = 19.178, *p* < 0.00, η^2^ = 0.444) and APP (F (1,24) = 45.204, *p* < 0.001, η^2^ = 0.653). The main effect of exercise was significant in p-TAU (F = 4.706,*p* = 0.040, η^2^ = 0.164) and APP (F (1,24) = 7.432, *p* = 0.012, η^2^ = 0.236). Then the simple effect test was conducted. Compared with the APP/PS1 group, HIIT significantly decreased the protein expression levels of p-TAU (​​F (1,24) = 13.247, *p* = 0.001, η^2^ = 0.356) and APP (F (1,24) = 11.646, *p* = 0.002, η^2^ = 0.327) (Figures 4A–C). The findings demonstrate that HIIT can mitigate hallmark AD pathology.

### 3.3 The effect of HIIT on the cerebral microangiogenesis in APP/PS1 mice

**FIGURE 5:**
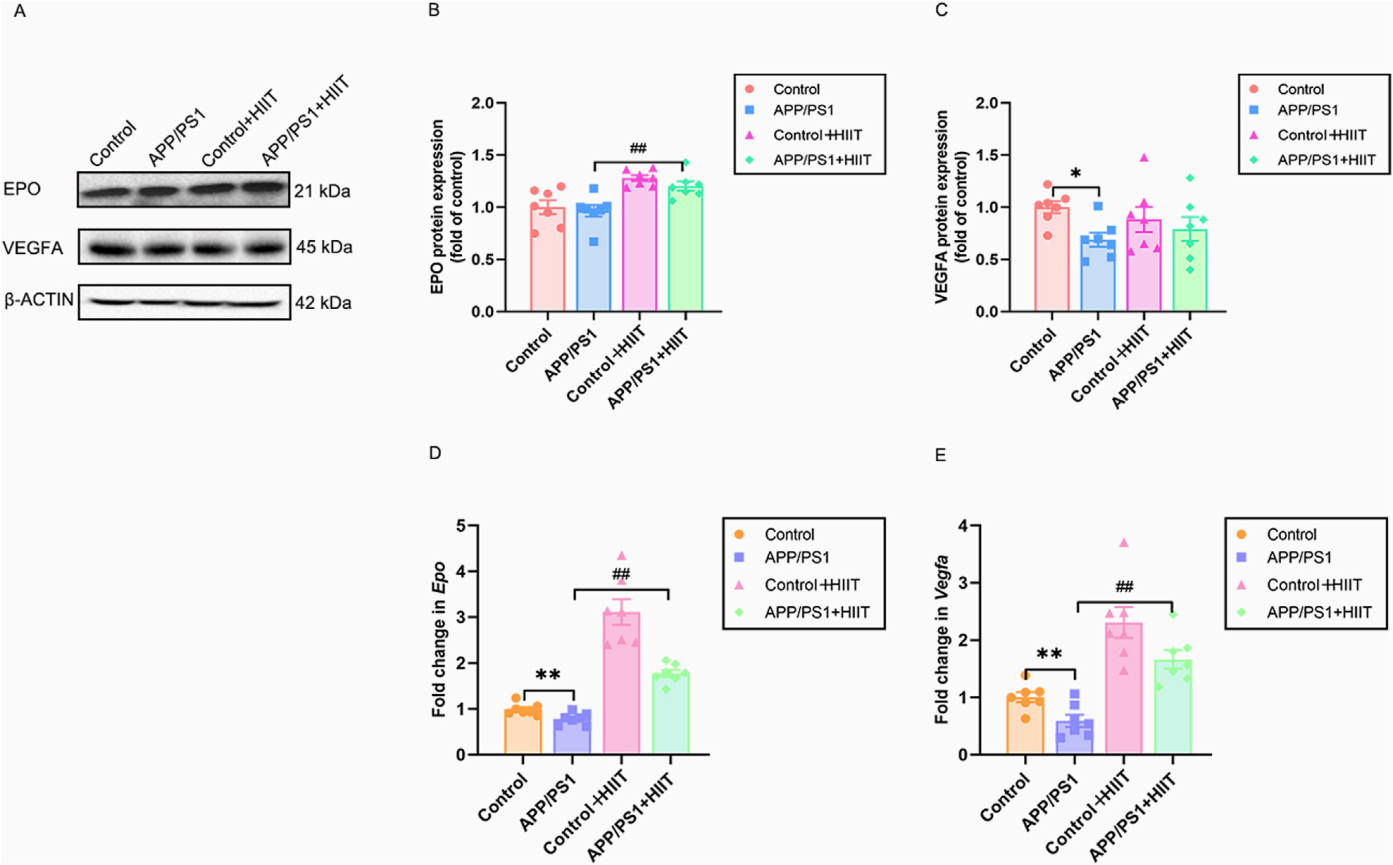
The effect of HIIT on the cerebral microangiogenesis in APP/PS1 mice. **(A)** the Western blot image of EPO and VEGFA. **(B)** quantification of EPO protein expression in each group. **(C)** quantification of VEGFA protein expression in each group. **(D)** the mRNA expression of *Epo* is shown for each of the four groups. **(E)** the mRNA expression of *Vegfa* is shown for each of the four groups. β-ACTIN and *Gapdh* are internal controls for quantification. Data displayed as mean ± SE (n = 7). ^*^: *p* < 0.05, *p* < 0.01 APP/PS1 group versus Control group; ^#^: *p* < 0.01 APP/PS1 + HIIT group versus APP/PS1 group.

Then changes of EPO and VEGF were detect to assess whether HIIT promotes cerebrovascular remodeling via angiogenesis under the condition of AD, depicted in Figure 5. In the protein levels, the genotype × exercise interaction was not significant in EPO (F (1,24) = 0.164, *p* = 0.690, η^2^ = 0.007) and VEGFA (F (1,24) = 1.379, *p* = 0.252, η^2^ = 0.054). The main effect of genotype was significant in VEGFA (F (1,24) = 4.623, *p* = 0.042, η^2^ = 0.162). The main effect of exercise was significant in EPO (F (1,24) = 25.341, *p* < 0.001, η^2^ = 0.514) (Figures 5B,C). In the mRNA levels, the genotype × exercise interaction was significant in *Epo* (F (1,24) = 14.207, *p* = 0.001, η^2^ = 0.372). The main effect of genotype was significant in *Vegfa* (F (1,24) = 9.617, *p* = 0.005, η^2^ = 0.286) and Epo (F (1,24) = 26.436, *p* < 0.001, η^2^ = 0.524). The main effect of exercise was significant in *Vegfa* (F (1,24) = 48.249, *p* < 0.001, η^2^ = 0.668) and *Epo* (F (1,24) = 108.010, *p* < 0.001, η^2^ = 0.818). Then the simple effect test was conducted for *Epo*. Compared with the APP/PS1 group, HIIT significantly increased the *Epo* mRNA levels (F (1,24) = 26.436, *p* < 0.001, η^2^ = 0.524) (Figures 5D,E), suggesting that HIIT potently stimulates pro-angiogenic signaling, particularly in APP/PS1 mice.

### 3.4 The effect of HIIT on the cerebrovascular regulation of APP/PS1 mice

**FIGURE 6:**
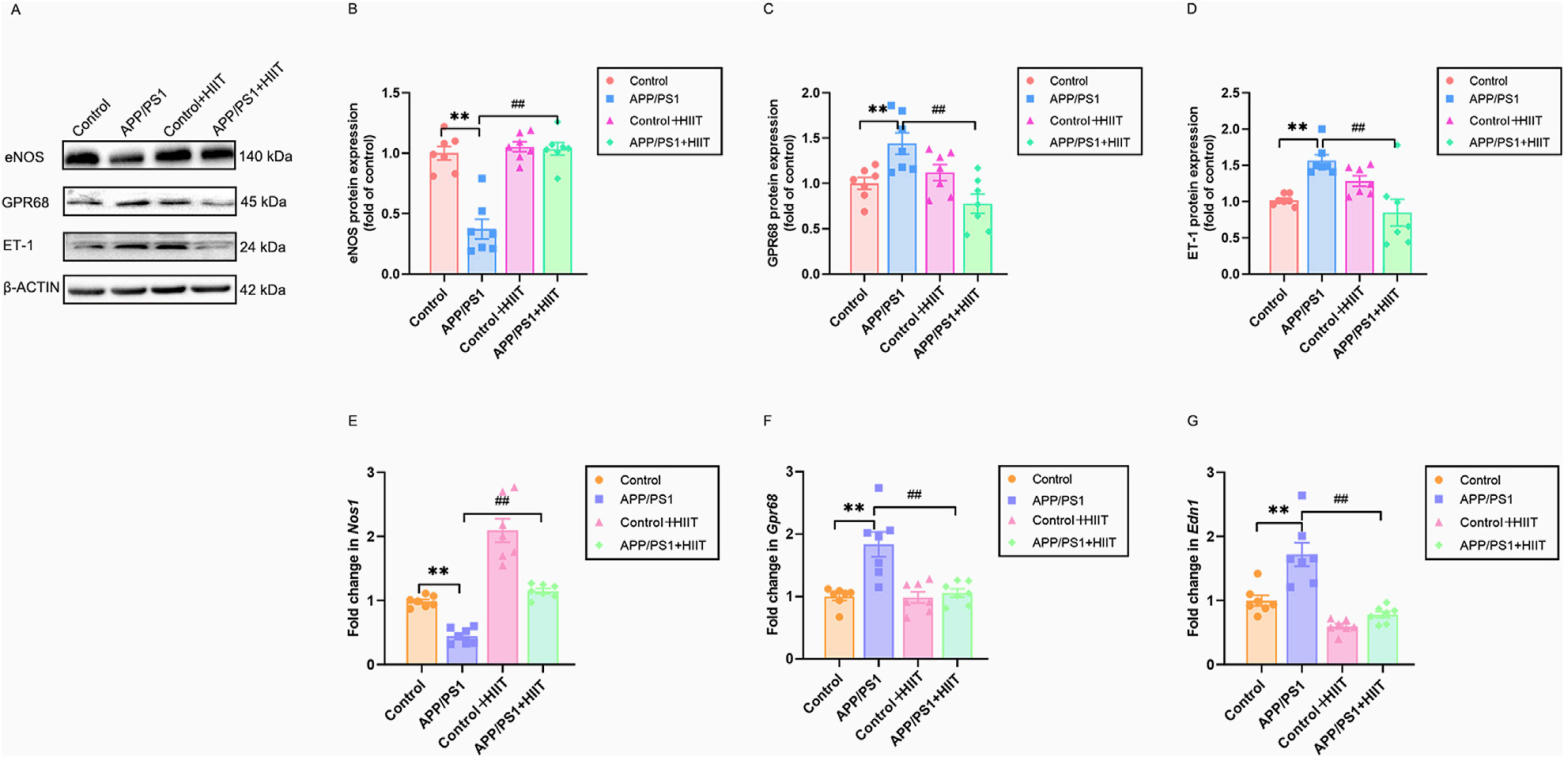
The effect of HIIT on the cerebrovascular regulation of APP/PS1 mice. **(A)** the Western blot image of eNOS, GPR68, and ET-1. **(B)** quantification of eNOS in each group. **(C)** quantification of GPR81 in each group. **(D)** quantification of ET-1 in each group. **(E)** the mRNA expression of *Nos1*. **(F)** the mRNA expression of *Gpr68*. **(G)** the mRNA expression of *Edn1*. β-ACTIN and *Gapdh* are internal controls for quantification. Data are displayed as mean ± SE (n = 7). ^*^: *p* < 0.01 APP/PS1 group versus Control group; ^#^: *p* < 0.01 APP/PS1 + HIIT group versus APP/PS1 group.

eNOS and GPR68 are vasodilatory factors and ET-1 is vasoconstrictor factors. We then examined these indicters to investigate how HIIT might improve cerebral blood flow. In the protein levels, the genotype × exercise interaction was significant in eNOS (F (1,24) = 26.193, *p* < 0.001, η^2^ = 0.522), GPR68 (F (1,24) = 16.324, *p* < 0.001, η^2^ = 0.405), and ET-1 (F (1,24) = 21.026, *p* < 0.001, η^2^ = 0.467). The main effect of genotype was significant in eNOS (F (1,24) = 29.220, *p* < 0.001, η^2^ = 0.549). While, the main effect of genotype was not significant in GPR68 (F (1,24) = 0.237, *p* = 0.631, η^2^ = 0.010) and ET-1 (F (1,24) = 0.256, *p* = 0.617, η^2^ = 0.011). The main effect of exercise was significant in eNOS (F (1,24) = 36.058, *p* < 0.001, η^2^ = 0.600), GPR68(F(1,24) = 7.849, *p* = 0.010, η^2^ = 0.246), and ET-1 F (1,24) = 4.518, *p* = 0.044, η^2^ = 0.158). Then the simple effect test was conducted. Compared with the APP/PS1 group, HIIT significantly increased the eNOS expresssion (F (1,24) = 61.858, *p* < 0.001, η^2^ = 0.720) and decreased the protein expression of GPR68 (F (1,24) = 23.406, *p* < 0.001, η^2^ = 0.494) and ET-1 (F (1,24) = 22.519, *p* < 0.001, η^2^ = 0.484) (Figures 6A–D). In the mRNA levels, genotype × exercise interaction was significant in *Nos*1 (F (1,24) = 4.373, *p* = 0.047, η^2^ = 0.154), *Gpr68* (F (1,24) = 10.656, *p* = 0.003, η^2^ = 0.307), *Edn1* (F (1,24) = 6.491, *p* = 0.018, η^2^ = 0.213). The main effect of genotype was significant in *Nos1* (F (1,24) = 57.828, *p* < 0.001, η^2^ = 0.707), *Gpr68* (F (1,24) = 14.774, *p* = 0.001, η^2^ = 0.381), and *Edn1* (F (1,24) = 18.494, *p* < 0.001, η^2^ = 0.435). The main effect of exercise was significant in Nos1 (F (1,24) = 85.963, *p* < 0.001, η^2^ = 0.782), *Gpr68* (F (1,24) = 11.134, *p* = 0.003, η^2^ = 0.317), and Edn1 (F (1,24) = 40.524, *p* < 0.001, η^2^ = 0.628). Then the simple effect test was conducted. Compared with the APP/PS1 group, HIIT significantly increased the Nos1 expresssion (F (1,24) = 57.828, *p* < 0.001, η^2^ = 0.707) and decreased the mRNA levels of *Gpr68* (F (1,24) = 23.406, *p* < 0.001, η^2^ = 0.494) and *Edn1* (F (1,24) = 18.494, *p* < 0.001, η^2^ = 0.435) (Figures 6E–G). These coordinated changes in vascular regulators suggest that HIIT promotes cerebrovascular dilation.

### 3.5 The effect of HIIT on the HIF-1α expression in APP/PS1 mice

**FIGURE 7:**
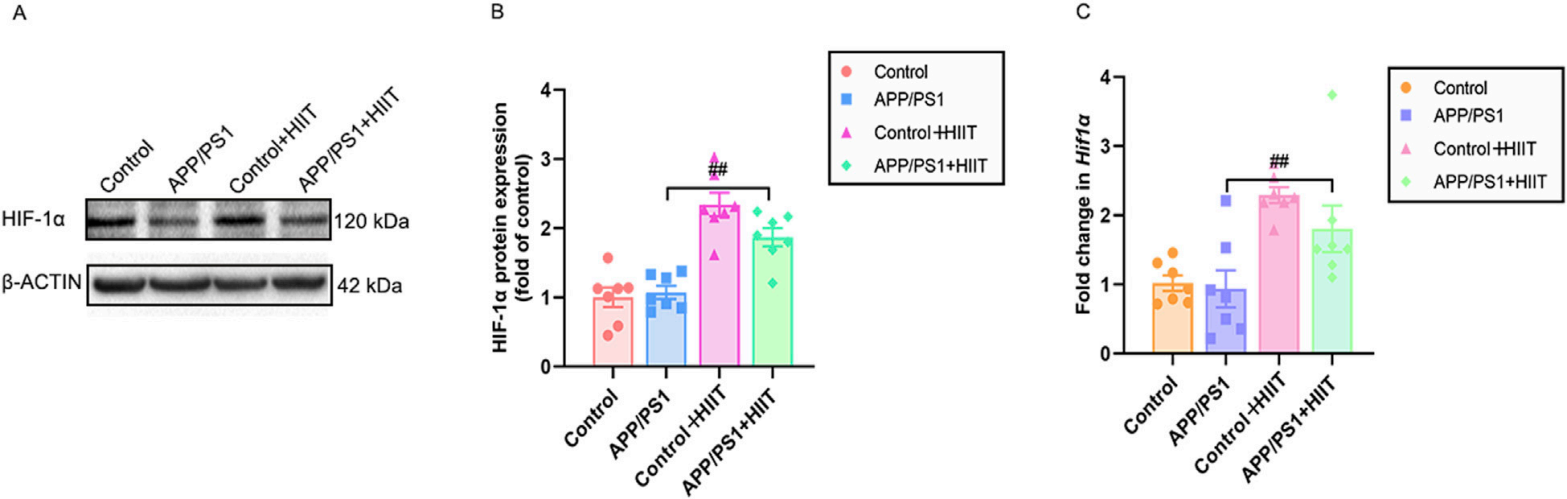
The effect of HIIT on the HIF-1α expression in APP/PS1 mice. **(A)** the Western blot image of HIF-1α. **(B)** quantification of HIF-1 α in each group. **(C)** the mRNA expression of *Hif-1α*. β-ACTIN and *Gapdh* are internal controls for quantification. Data displayed as mean ± SE (n = 7). ^#^: *p* < 0.05, *p* < 0.01 APP/PS1 + HIIT group versus APP/PS1 group.

Since HIF-1α is a potential upstream regulator of the observed vascular changes, ​​we detected​​ its protein and mRNA expression, as shown in Figure 7. Genotype × exercise interaction was not significant in protein levels (F (1,24) = 3.863, *p* = 0.061, η^2^ = 0.139) and mRNA levels (F (1,24) = 0.779, *p* = 0.386, η^2^ = 0.031). The main effect of genotype was not significant in protein levels (F (1,24) = 2.127, *p* = 0.158, η^2^ = 0.081) and mRNA levels (F (1,24) = 1.531, *p* = 0.228, η^2^ = 0.060). However, the main effect of exercise was significant in protein levels (F (1,24) = 59.871, *p* < 0.001, η^2^ = 0.714) and mRNA levels (F (1,24) = 21.687, *p* < 0.001, η^2^ = 0.475) (Figures 7A–C). The result suggests that HIIT-induced HIF-1α activation may represent a fundamental mechanism driving the observed vascular improvements, independent of AD pathology.

## 4 Discussion

In AD, TAU becomes abnormally aggregated into amyloid filaments featuring cross-β structures, which, along with diminished clearance in the brain, contributes to the process known as amyloidogenesis-the formation of amyloid fibrils (Parra Bravo et al., 2024; Annadurai et al., 2021). The phosphorylation process plays an essential role in TAU protein’s physiological function, which is related to amyloid metabolism and synaptic integrity (Wesenhagen et al., 2022). APP is hydrolyzed to generate Aβ, a key component of amyloid plaques (Akasaka-Manya and Manya, 2020), which is a pathological feature of AD. In our results, we found that APP/PS1 significantly increased both the TAU protein phosphorylation and APP levels. These results imply that hyperphosphorylated TAU promotes amyloid plaques and neurofibrillary tangles formation, which deteriorates AD development (Naseri et al., 2019). Meanwhile, overproducing APP fragments triggers typical Aβ pathology, neuroinflammation, and memory impairment in an age-dependent manner (Saito et al., 2014). Moreover, accumulation of mutant APP has been reported to be responsible for reduced MAP2, dendritic spines, and hippocampal-based impaired learning and memory in 12-month-old mice (Manczak et al., 2018). These results hint that excessive P-TAU and APP drive the pathology of AD and contribute to the reduction of synaptic strength, synaptic loss, and neurodegeneration in this AD model. In comparison, HIIT inhibited the expression of p-TAU and APP in the hippocampus of APP/PS1 mice, indicating its capacity to mitigate AD-like pathology and potentially improve cognitive function​​.

Given that TAU and APP aggregation correlate with the severity of cognitive impairment in AD patients (Stevens and Brown, 2015), we assessed memory in mice. We found the increase of reference memory error and the working memory errors in the APP/PS1 mice, suggesting the short- and long-term memory impairment. BDNF critically supports hippocampal long-term potentiation and synaptic efficacy, which ​​is positively associated with​​ memory (Lu et al., 2014). We also detected the mRNA expression of *Bndf* to evaluate whether the cognitive impairment involved molecular levels. *​​Bdnf​​* mRNA levels ​​were reduced​​ in APP/PS1 mice versus wild-type littermates, suggesting neuroplasticity impairment ​​triggered by​​ the APP/PS1 mutation. In contrast,​​ HIIT significantly reduced the reference memory error and enhanced the mRNA expression of *Bndf* in APP/PS1 mice, ​​indicating​​ potential recovery of cognitive function and neuroplasticity. However, HIIT had no significant effect on working memory errors​​. This limited effect may be attributed to the duration of HIIT in our study. For example, longer interventions of HIIT (e.g., 8-week (Feng et al., 2023) or 10-week (Liu et al., 2022)) have shown broader cognitive benefits in AD models, such as learning and memory and a desire for exploration.

EPO is linked to cerebrovascular events and ameliorates cognitive deficits by reducing oxidative stress, mitigating inflammatory responses, enhancing ATP generation, activating serotonergic signaling pathways, and facilitating the clearance of Aβ plaques in the hippocampus of AD mice (Dara et al., 2019; Shim et al., 2022). It was reported that there is a decline in the hippocampus of aging rats (Li et al., 2016). Our results indicated that the mRNA expression of *Epo* decreased, whereas there were no changes in the protein levels in APP/PS1 mice compared to the wildtype. We speculated that the inconsistent results could be explained by the following factors: the maintenance of EPO protein levels may represent an endogenous compensation or protection mechanism during AD pathology; the pathological environment of AD (such as Aβ accumulation) may inhibit protein degradation pathways, leading to a reduced turnover rate of EPO protein, thereby maintaining its steady-state levels even when mRNA levels decrease; The inconsistency between protein and gene transcription suggests that the regulation of EPO expression may occur at the post-transcriptional or post-translational levels. After a 6-week HIIT intervention, both the mRNA and protein expression of EPO were significantly increased in the APP/PS1 + HIIT group compared to the APP/PS1 group. The results demonstrated that HIIT can effectively reverse the pathological inhibition of EPO expression in the APP/PS1 mouse model, simultaneously increasing its mRNA transcription levels and protein synthesis efficiency.

VEGF is also a classical vasoactive molecule involved in neurogenesis (During and Cao, 2006). Aberrant VEGF signaling may lead to impaired brain capillary function and reduced blood flow, contributing to cognitive decline (Ali and Bracko, 2022). Our results indicated that both mRNA and protein expression of VEGF were reduced in APP/PS1 mice, suggesting impaired cerebrovascular function. These findings were consistent with those of Tsartsalis et al., who observed decreased immunostaining of VEGF in vascular endothelial cells of AD patients, along with disorders of angiogenesis and compromised BBB integrity (Tsartsalis et al., 2024). Previous studies have demonstrated that exercise-induced hippocampal neurogenesis (Fabel et al., 2003) and cerebral microangiogenesis (Morland et al., 2017; Ding et al., 2006) depended on the VEGF. However, our findings indicate that only mRNA expression increased in the AD-trained mice. This divergence between transcriptional activation and translational output suggests that post-transcriptional regulatory mechanisms might predominantly control VEGF homeostasis in the AD brain during exercise intervention. Moreover, the discrepancy could also stem from differences in exercise patterns, duration, intensity, and the organism’s physiological state (Vital et al., 2014). In summary, these findings imply that HIIT improves cerebrovascular remodeling in the pathological state of AD by mainly stimulating EPO.

Vasculopathy in AD brain regions, particularly in white matter vessels, correlates with reduced eNOS expression levels in cerebral vessels (de la Monte et al., 2000). Our findings revealed that protein levels of eNOS and mRNA levels of *Nos1* were decreased in APP/PS1 mice, indicating possible vasculopathy in the hippocampal region. Concurrently, the reduced production of NO, the end-product of eNOS, could exacerbate endothelial inflammation and vasoconstriction, resulting in damage (!!! INVALID CITATION), implying that APP/PS1 mutation may lead to reduced cerebral perfusion in the rodent model. Supporting this notion, studies in eNOS knockout mice showed an increased perfusion response to whisker stimulations in the barrel cortex alongside reduced glutamine levels in the frontal cortex, hippocampus, parahippocampal region, and cerebellum. These changes led to classical AD-like pathological manifestations, including Aβ accumulation and memory deficits (Hariharan et al., 2019). This is because chronic reduced cerebrovascular eNOS and NO could impair vasodilation responses and diminish the capacity to remove respiratory waste products and toxins from the extracellular space (de la Monte et al., 2000). Consequently, the impairment in working and reference memory observed in APP/PS1 mice is likely associated with their reduced eNOS levels. Following the exercise intervention, the mRNA expression of *Nos1* significantly increased in the wild-type mice. Moreover, the expression levels of eNOS and *Nos1* in the APP/PS1 mice were restored or nearly reached those seen in normal wild-type mice. Previous studies have shown that running exercise markedly enhances eNOS activity in the rat cortex, resulting in improved cerebrovascular function. This includes upregulation of proangiogenic factors, as well as increases in the length, volume, and surface area of capillaries, and a decrease in antiangiogenic factors (Zang et al., 2023). Hence, our results hinted that this exercise regimen promoted eNOS expression to facilitate vasodilation of hippocampal cerebrovascular structures, thereby improving perfusion.

In the stroke brain, overexpression of GPR68 exerted neuroprotection and was able to alleviate the neuronal damage via inhibiting the protein kinase C (PKC) activation (Wang et al., 2020). In our study, however, ​​we observed abnormally elevated levels of GPR68 protein and mRNA in APP/PS1 mice, suggesting that its upregulation may represent a compensatory protective mechanism against neuronal damage.​​ Following exercise intervention, GPR68 levels returned to those of the control group. We speculated that HIIT ameliorates AD pathology by mitigating stressors such as acidosis and inflammation (Yu et al., 2025), which would otherwise sustain compensatory GPR68 upregulation.​​ This implies that prolonged GPR68 activation is unnecessary, ​​given its dual roles: activation in acidic microenvironments (Ludwig et al., 2003) and induction of pro-inflammatory cytokine release (Ichimonji et al., 2010). Additionally, HIIT may provide more potent neuroprotective molecules like BDNF, potentially restoring GPR68 expression to physiological levels or reducing its functional demand.

The elevated ET-1 levels have been reported to increase BBB permeability and correlate with the degree of cerebral hypoperfusion in patients with AD and related dementia (D'Orleans-Juste et al., 2019). Our results showed that both protein and mRNA expression of ET-1 were significantly increased in the hippocampus of APP/PS1 mice, implying enhanced cerebral vasoconstriction in AD. This finding aligns with the established role of vascular dysregulation in AD pathogenesis. The elevated ET-1 expression likely reflects endothelial dysfunction, a recognized hallmark of AD progression. Furthermore, we found that HIIT reduced the protein levels of ET-1 and the mRNA expression of *Edn1* compared to the APP/PS1 mice, suggesting that physical activity may inhibit ET-1 release, thereby improving vascular perfusion. This mechanism may ​​promote cerebrovascular dilation and enhance CBF, consequently optimizing oxygen and nutrient delivery to brain tissues​​. In summary, HIIT contributes to cerebrovascular repair through positive regulation of vascular function, ultimately ameliorating brain health in AD.

Pre-clinical findings indicated an important role for the endogenous oxygen sensing machinery, particularly HIF and its target genes, in proper cognitive function (Gruneberg et al., 2016). During the development of AD pathology, dysregulated HIF-1α activation may exacerbate cerebral hypoperfusion (Porel et al., 2024). HIF-1α is implicated in the production of pro-inflammatory cytokines and Aβ, further contributing to neuronal damage (Alexander et al., 2022; Jung et al., 2023). Conversely, HIF-1α may support synaptic plasticity and neuronal survival by counteracting the toxic effects of Aβ and inhibiting p-TAU (Wang et al., 2021). In our study, we found no significant difference in HIF-1α protein or mRNA expression in the hippocampus between control mice and APP/PS1 mice. Similar to our results, Maria et al. found no changes in the mRNA expression of HIF-1α in APP/PS1 mice from 3 to 12 months of age (de Lemos et al., 2013). Sabrina et al. also reported unchanged HIF-1α expression in nuclear extracts from the cortex of 5xFAD transgenic mice (Petralla et al., 2024). This lack of change may be attributed to several factors: (1) Early hypoxia in AD might be compensated by alternative pathways, preventing significant fluctuations in HIF-1α levels; (2) the regulation of HIF-1α through post-translational modifications, such as acetylation, provides another layer of complexity in its stabilization (3) Differences in AD mouse models (e.g., specific mutations and disease stages examined); may influence whether or not a robust hypoxia response is triggered. For example, one potential compensatory mechanism involves the interaction between NO and HIF-1α, which can prevent HIF-1α degradation in astrocytes, initiating a response to hypoxia (Shi et al., 2016). Furthermore, chronic hypoxia in AD brains can promote inflammasome formation (Jung et al., 2023), which in turn may maintain HIF-1α expression, potentially establishing a detrimental feedback loop contributing to vascular damage. Furthermore, acetylation can stabilize HIF-1α protein levels without affecting mRNA expression, enhancing its stability during inflammation. This suggests that similar mechanisms may function under hypoxic conditions in AD to maintain HIF-1α levels (Chen Q. et al., 2022). Consistent with the notion of model-dependent effects, HIF-1α conversely upregulation has been reported in the hippocampus and cortex of other AD models, such as SAMP8 (Shang et al., 2024) and 3x-Tg AD mice (Jung et al., 2023).

Interestingly, prior research has demonstrated that exercise can induce tissue hypoxia in organs like the heart (Xu et al., 2024) and small intestine (Wu et al., 2020) for metabolic adaptation. There is limited evidence concerning the role of HIF-1α in the brain during exercise. Our findings indicated that HIIT significantly enhanced both protein and mRNA expression of HIF-1α in the hippocampus of APP/PS1 training mice compared to sedentary APP/PS1 controls. This HIIT-induced upregulation of HIF-1α may indicate an enhanced metabolic adaptation to hypoxia in the context of AD. On the one hand, elevated HIF-1α can promote the expression of factors essential for angiogenesis (e.g., EPO, VEGF) and regulate cerebrovascular function (e.g., eNOS and ET-1), thereby improving CBF and oxygen delivery to meet metabolic demands, particularly under hypoxic stress. On the other hand, upregulating HIF-1α by exercise may promote neuroprotection in APP/PS1 mice, potentially by enhancing BDNF expression and rescuing synaptic loss (Guo et al., 2015).

## 5 Conclusion

Physical activity, particularly HIIT, has been shown to induce beneficial changes that are essential for maintaining cognitive function and overall brain health. These benefits arise from various mechanisms, including improving cerebrovascular function like enhanced blood oxygen supply and cerebral perfusion. Our research first observed that 6-week HIIT was conducive to cerebrovascular health through promoting cerebral microangiogenesis and modulating cerebrovascular function. It also enhanced the HIF-1α expression to modulate the hypoxic metabolism environment for AD, which may act as an upstream regulator​​ of vascular factor release and angiogenesis signaling. Further mechanistic studies are needed to confirm its regulatory role. ​​Collectively, these HIIT-induced adaptations​​ ultimately contribute to a reduction in APP and TAU protein levels in AD. These findings highlight HIIT as a promising non-pharmacological strategy for mitigating cerebrovascular dysfunction in AD models. However, clinical application requires validation in human studies.

## 6 Limitations

Firstly, the number of animals in each group in this experiment is relatively small. Our animal model only applied to the female mice, which lacked evidence of the role of HIIT on the male AD mice. However, this limitation does not affect the overall conclusion. Future studies should aim to expand the sample size for each group to further ensure the vascular protective effect of HIIT on the AD of different genders. Secondly, we adopted the 6-week duration of HIIT, which was insufficient to trigger an obvious long-term exercise dose effect. Further study may include multiple exercise patterns to compare the differences between those exercises in AD treatment as possible. Thirdly, in this study, we identified for the first time that HIIT promotes brain health in AD by enhancing cerebral blood vessel function. The use of neuroimaging technologies and histological experiment to observe cerebrovascular changes will be necessary to further elucidate the effects of HIIT on AD. ​​Lack of direct vascular and Aβ morphological data weakens mechanistic conclusions.​​ Finally, although the HIF-1α levels in the hippocampus did not significantly change in AD mice, they were enhanced by exercise. This study also did not establish a causal relationship between the exercise-induced increase in HIF-1α expression and various vascular factors associated with the pathological changes in AD. Given the potential association between HIF-1α and these factors, it is essential to employ genetic tools to knock out or knock down HIF-1α in rodent models. This approach could clarify the regulatory effects of HIF-1α on cerebral vascular metabolism in AD, providing deeper insights into the molecular mechanisms by which exercise improves cerebrovascular function and identifying molecular targets for exercise interventions. Notably, we selected 4-month-old APP/PS1 mice as a model for the early stages of AD. Further research is necessary to investigate the effects of HIIT on AD pathology across both early and late stages, as this may help to elucidate the underlying mechanisms through which exercise prevents and treats AD, ultimately aiding in the formulation of specific exercise strategies tailored for the disease.

## Data Availability

The original contributions presented in the study are included in the article/supplementary material, further inquiries can be directed to the corresponding author.
